# Draft genome sequence data on *Streptomyces salinarius* MPA0124 isolated from coastal sediments of Pondicherry, India

**DOI:** 10.1016/j.dib.2025.112401

**Published:** 2025-12-16

**Authors:** Munisamy Prathaban, Murugesan Sobanaa, S. Hari Krishna Kumar, Ragothaman Prathiviraj, George Seghal Kiran, Joseph Selvin, Laurent Dufossé

**Affiliations:** a Carbon Capture and Utilization Laboratory, Department of Microbiology, Pondicherry University, Pondicherry 605014, India; b Microbial Genomics Laboratory, Department of Microbiology, Pondicherry University, Pondicherry 605014, India; c Division of Bioinformatics and Systems Biology, Department of Biosciences, Rajagiri College of Social Sciences (Autonomous), Rajagiri P.O., Kalamassery, Kochi 683104, India; d Department of Food Science and Nutrition, Pondicherry University, Pondicherry 605014, India; e CHEMBIOPRO Lab (Chemistry and Biotechnology of Natural Products), University of La Réunion, ESIROI Agroalimentaire, 97744 Saint-Denis Cedex 9, Réunion, France

**Keywords:** Biosaline biotechnology, Carbohydrate-active enzymes (CAZymes), Coastal sediments, Draft genome, *Streptomyces salinarius*

## Abstract

*Streptomyces salinarius* MPA0124, a halotolerant actinobacterium, was isolated from coastal sediments of Pondicherry, India, using Bennett’s agar supplemented with 5 % NaCl. The draft genome, sequenced using the Illumina HiSeq platform, comprises 8.01 Mb across 122 contigs with a GC content of 72.22 %. Genome annotation predicted 7548 coding sequences, 70 tRNAs, and 4 rRNAs, indicating a metabolically diverse genetic repertoire. Functional analysis revealed a broad array of carbohydrate-active enzymes (CAZymes) involved in cellulose, hemicellulose, pectin, and lignin degradation, highlighting the strain’s role in polysaccharide turnover and carbon recycling in saline ecosystems. Phylogenomic analysis clustered *S. salinarius* MPA0124 closely with *S. salinarius* SS06011 and *S. ardesiacus* NBRC15402, confirming its taxonomic placement within the *Streptomyces* genus. The genomic dataset enriches the current knowledge of marine-derived *Streptomyces* and underscores its potential for biotechnological applications in biomass conversion, enzyme discovery, and biosaline agriculture. The genome sequence has been deposited in NCBI under the accession number NZ_JBJDRC000000000.1.

Specification Table

**Table:** 

Subject	Applied Microbiology
Specific subject area	Genomics
Type of data	Whole genome sequence of *S. salinarius* MPA0124 raw and analyzed, represented as tables and figures
Data collection	Genomic DNA was isolated using a DNA extraction kit from HiMedia, India. The whole genome was sequenced at Macrogen Inc. (Seoul, South Korea) using the Illumina HiSeq PE150 platform. The quality check was done in FastQC software version 0.11.9, to check the adapter sequence and low-quality regions in the sequence. The Fastp tool was used to remove the adapter region. After pre-processing, the sequence was assembled using the Megahit assembler and the genome statistics were assessed by the Quast tool. The assembled genome was visualized using Proksee server, while genome annotation and gene prediction were performed with Prokka v1.14.6.
Data source location	*S. salinarius* strain MPA0124 was isolated from sediment samples taken from the intertidal region of the Pondicherry coast in India (11.900476° N, 79.812477° E).
Data accessibility	Whole Genome data was deposited in the National Center for Biotechnology Information (NCBI) Genbank database with the following BioProject ID: PRJNA224116. The deposited draft genome data available at https://www.ncbi.nlm.nih.gov/nuccore/NZ_JBJDRC000000000.1
Related research article	None

## 1 Value of the Data

• The *S. salinarius* MPA0124 draft genome sequence provides valuable insights into the genetic potential of coastal actinobacteria adapted to saline environments.
• Researchers working in marine microbiology and environmental biotechnology, as well as in the discovery of natural products, might find this genomic data beneficial for investigating enzymes of industrial significance, bioactive secondary metabolites, and mechanisms of stress tolerance.
• The genome sequence data can facilitate comparative genomic studies among *Streptomyces* species from diverse habitats, aiding in the understanding of evolutionary adaptation, gene cluster diversity, and ecological functions.
• This dataset contributes to the genomic repository of marine-derived *Streptomyces*, supporting future applications in biotechnology, pharmaceuticals, and environmental sustainability.

## 2 Background

*S. salinarius* is a Gram-positive, filamentous actinobacterium commonly found in saline and marine environments. Members of the genus *Streptomyces* are renowned for their ability to produce a wide range of secondary metabolites, including antibiotics, enzymes, and other bioactive compounds with significant industrial, pharmaceutical and agricultural importance [1]. Coastal and marine sediments represent unique ecological niches where salinity, organic matter, and microbial interactions drive the evolution of metabolically versatile microorganisms [2]. The adaptation of *Streptomyces* species to saline habitats involves specialized genomic features that enable osmotic tolerance, efficient nutrient utilization, and biosynthesis of novel metabolites [3]. The present study reports the first draft genome of a marine sediment-derived strain, *S. salinarius* MPA0124, from the Indian coast. Comparative genomic analysis indicates distinct environmental adaptations, including a higher diversity of CAZymes and stress-response genes, underscoring its potential for biosaline biotechnology and marine biomass valorization.

## 3 Data Description

**Fig. 1:**
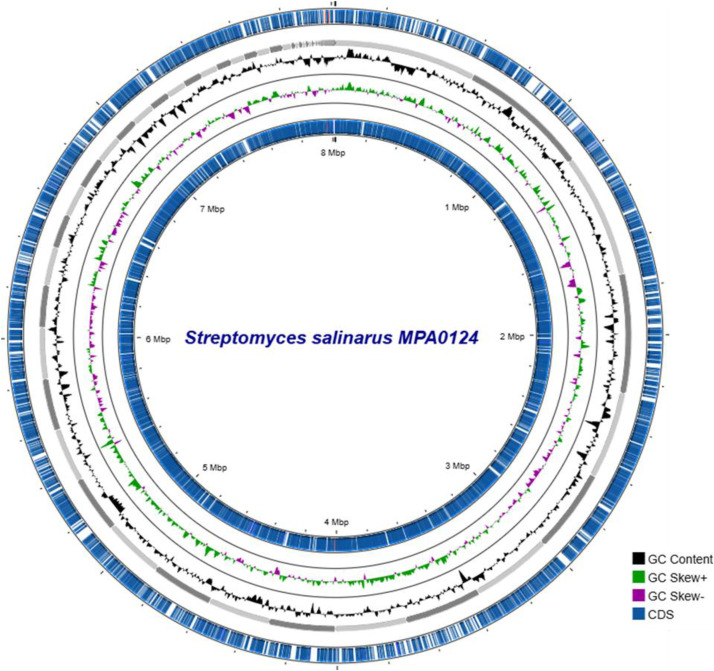
Circos plot representation of the *Streptomyces salinarius* MPA0124 draft genome. The outermost circle depicts coding DNA sequences (CDS) annotated on both forward and reverse strands, with key genes and functional elements labeled in blue. The inner circles represent genomic features: GC content variation (black), GC skew (+) in green, and GC skew (−) in magenta. The genome map illustrates the overall organization and compositional features across the 8 Mbp genome with a GC content of 72.22 %.

The draft genome sequence of *S. salinarius* strain MPA0124, isolated from coastal sediments of Pondicherry, India, was assembled into 122 contigs, yielding a total genome length of 8015,236 bp with an overall GC content of 72.22 % (Fig. 1). The assembly exhibited a contig N50 value of 313,301 bp and an L50 of 9, indicating a high-quality draft genome. A total of 7548 coding DNA sequences (CDS) were predicted, along with 70 tRNA genes and 4 rRNA genes, suggesting a complete set of essential genetic components (Table 1). Based on the whole-genome comparison, *S. salinarius* MPA0124 exhibited an ANI value of 95.62 % and a dDDH value of 63.8 % against the type strain *S. salinarius* SS06011, both below the standard species demarcation thresholds (96 % ANI and 70 % dDDH). These results suggest that MPA0124 represents a genomically distinct variant, potentially a novel subspecies or genomospecies within the *S. salinarius* clade, possibly reflecting ecological adaptation to marine coastal sediments. The genome statistics collectively indicate that *S. salinarius* MPA0124 possesses a complex and metabolically versatile genome, typical of marine-derived *Streptomyces* species, supporting its potential for secondary metabolite biosynthesis and environmental adaptation in saline habitats.

**Table 1:** Genome statistics of *S. salinarius* MPA0124.

Features	Count
Contigs	122
GC Content	72.22
Genome Length	8015,236 bp
Contig L50	9
Contig N50	313,301
CDS	7548
tRNA	70
rRNA	4
Hypothetical proteins	1947

**Fig. 2:**
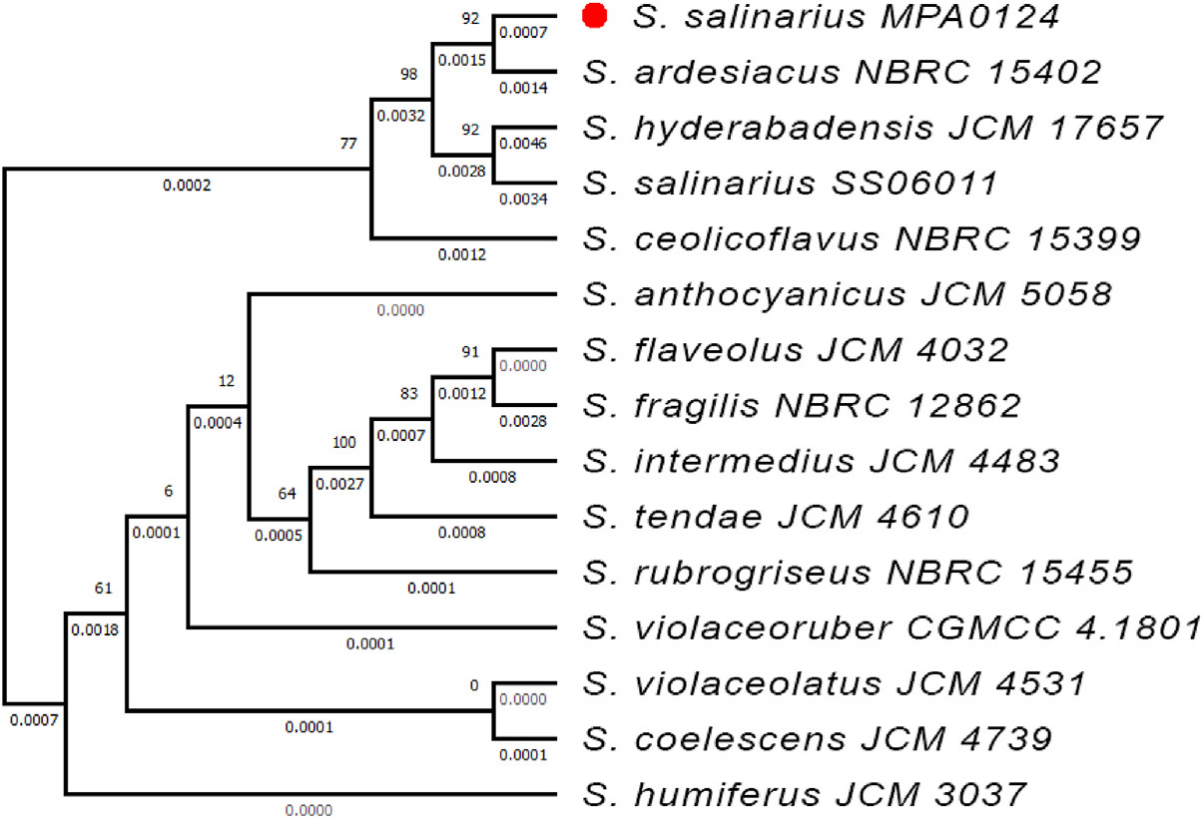
Phylogenetic tree based on 16S rRNA gene sequences showing the evolutionary relationship of *Streptomyces salinarius* MPA0124 (marked with a red circle) with closely related Streptomyces species. The tree was constructed using the neighbor-joining method, and bootstrap values (expressed as percentages of 1000 replications) are shown at the branch nodes. Evolutionary distances are indicated as substitutions per nucleotide position.

**Fig. 3:**
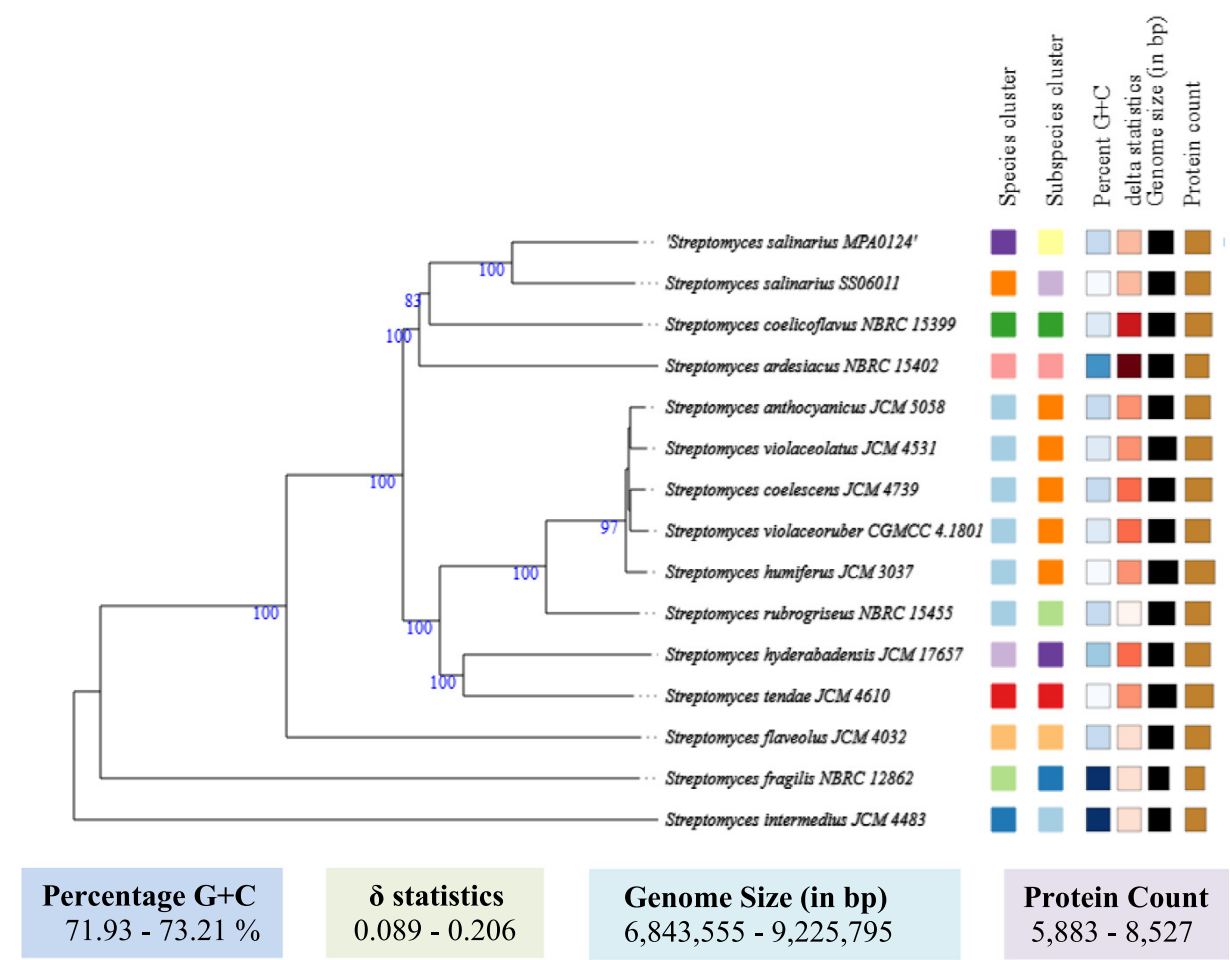
A phylogenomic tree was constructed using FastME version 2.1.6.1 based on GBDP distances derived from genome sequences. The branch lengths are represented according to the GBDP distance formula d5. Values above the branches indicate GBDP pseudo-bootstrap support greater than 60 % from 100 iterations, resulting in an average branch support of 82.6 %. The tree was centered at the midpoint for rooting.

The phylogenetic tree shows *that S. salinarius* MPA0124 (red-marked) clusters closely with *S. ardesiacus* NBRC15402, *S. hyderabadensis* JCM17657, and *S. salinarius* SS06011, indicating a close evolutionary relationship among these strains (Fig. 2). The high bootstrap values support the robustness of this clade within the *Streptomyces* genus. The phylogenomic tree demonstrates that the target strain *S. salinarius* MPA0124 is closely related to *S. salinarius* SS06011, validating species-level classification. The strong bootstrap values in most branches increase confidence in the reliability of these evolutionary relationships. The clustering with other *Streptomyces* species (purple block) shows evolutionary relatedness within the genus. *S. intermedius* JCM 4483 is found to be an ancestor for the target genome *S. salinarius* MPA0124, as depicted in Fig. 3.

Functional genome annotation of *S. salinarius* MPA0124 revealed the presence of multiple genes encoding carbohydrate-active enzymes (CAZymes) involved in lignocellulosic biomass degradation. The strain harbors a diverse set of cellulolytic, hemicellulolytic, pectinolytic, and ligninolytic enzymes, highlighting its potential role in polysaccharide decomposition and carbon recycling in saline coastal environments (Table 2). A total of 28 genes were identified under the cellulase group, including endoglucanases (2 genes), exoglucanase (1 gene), cellulases (4 genes), β-glucosidases (18 genes), α-glucosidase (1 gene), oligo-1,6-glucosidase (1 gene), and β-galactosidase (1 gene), suggesting a robust cellulose hydrolysis capability. The hemicellulase group comprised xylanases (8 genes), arabinose-degrading enzymes (6 genes), and mannose-related enzymes (2 genes), which collectively contribute to hemicellulose breakdown.

**Table 2:** Distribution of genes encoding carbohydrate-degrading enzymes identified in the genome of *Streptomyces salinarius* MPA0124.

Enzyme	Subtype	No. of Genes
Cellulase	Endoglucanase	2
	Exoglucanase	1
	Cellulase	4
	β-glucosidase	18
	α-glucosidase	1
	Oligo-1,6-glucosidase	1
	β-galactosidase	1
Hemicellulase	Xylanase	8
	Arabinose	6
	Mannose	2
Pectinase	Pectinase	2
	Pectinesterase	3
Ligninase	Laccase	2
	Polyphenol oxidase	1

In addition, pectinase (2 genes) and pectinesterase (3 genes) were detected, indicating the ability to degrade complex pectic substances. The presence of laccase (2 genes) and polyphenol oxidase (1 gene) further supports the strain’s potential for lignin modification and oxidative degradation. Overall, the genomic profile demonstrates that *S. salinarius* MPA0124 possesses an extensive repertoire of carbohydrate-degrading enzymes, underscoring its ecological and biotechnological significance in biomass conversion and marine bioprocessing applications. The presence of a diverse repertoire of cellulases, xylanases, and lignin-modifying enzymes in *S. salinarius* MPA0124 is consistent with reports from other *Streptomyces* species known for efficient lignocellulose degradation. Previous studies have demonstrated that marine and soil-derived *Streptomyces* strains harbor abundant CAZyme families, including glycoside hydrolases (GHs), carbohydrate esterases (CEs), and auxiliary activity (AA) enzymes, which contribute to biomass decomposition and carbon cycling in natural ecosystems [[4], [5], [6]]. These comparative insights validate the genome-based predictions observed in *S. salinarius* MPA0124 and highlight its potential role in polysaccharide turnover in saline coastal sediments.

## 4 Experimental Designs, Materials and Methods

*S. salinarius* strain MPA0124 was isolated from sediment samples taken from the intertidal region of the Pondicherry coast in India (11.900476° N, 79.812477° E). Using the conventional isolation method, the strain was isolated by culturing it on Bennett’s agar fortified with 5 % (w/v) sodium chloride. The resulting single colony was then re-cultured on Bennett’s agar plates and incubated for 96 h at a temperature of 28 ± 2 °C.

### 4.1 Genomic DNA extraction from Streptomyces culture

For the extraction of DNA, strain MPA0124 was grown in Bennett’s broth with 5 % NaCl at 30 °C for 72 h. The cells were separated from the broth through centrifugation, and DNA was isolated using a DNA extraction kit from HiMedia, India.

### 4.2 Whole genome sequencing and assembly

The whole genome of MPA012 was sequenced at Macrogen Inc. (Seoul, South Korea) using the Illumina HiSeq PE150 platform. The whole-genome sequencing of *S. salinarius* MPA0124 yielded a total sequence of 2941,622 in both forward and reverse directions. The quality check was done in FastQC software version 0.11.9 [7]. To check the adapter sequence and low-quality region in the sequence, the Fastp tool [8] was used to remove the adapter region. After pre-processing, the sequence was assembled using the Megahit assembler [9] and the genome statistics were assessed by the Quast tool [10]. The assembled genome circular plot was constructed using the Proksee server (https://proksee.ca/) and the assembled genome was annotated and gene predictions were carried out using Prokka version 1.14.6.

### 4.3 Phylogenetic tree construction and phylogenomic assessment

The genome sequence data were submitted to the Type (Strain) Genome Server (TYGS; https://tygs.dsmz.de) for conducting a taxonomic evaluation using the complete genome [11], using the most recent methodological advancements [[12], [13], [14]]. Data regarding nomenclature, synonymy, and associated taxonomic information were obtained from the List of Prokaryotic names with Standing in Nomenclature (LPSN; https://lpsn.dsmz.de) [12]. The closest type strain genomes were identified through two methods: (i) comparing user genomes against all type strain genomes in the TYGS database utilizing the MASH algorithm to select the ten with the smallest MASH distances [15]; and (ii) extracting 16S rDNA sequences with RNAmmer [16], followed by performing BLAST analysis [17] against 23,787 type strains in the TYGS database. The top 50 matches (based on bitscore) were then used to estimate distances accurately via Genome BLAST Distance Phylogeny (GBDP) employing the ‘coverage’ algorithm and distance formula d5 [18], allowing for the identification of the ten closest type strains. Intergenomic distances were calculated with the “trimming” algorithm in combination with the d5 distance formula [18]. Each pairwise comparison included 100 replicated distance calculations. Digital DNA-DNA hybridization (dDDH) estimates and their corresponding confidence intervals were produced according to the recommended settings of the GGDC 4.0 platform [18,19]. Average Nucleotide Identity (ANI) was calculated using validated algorithms optimized for large-scale genomic comparisons [20].

## Limitations

Not applicable.

## Ethics Statement

This study did not involve any experiments on human participants or animals. All data were obtained through computational analyses. Hence, ethical approval was not required for this work.

## CRediT Author Statement

**Prathaban Munisamy:** Validation, Conceptualization, Methodology, Writing – original draft, Writing – review & editing; **Sobanaa Murugesan:** Writing – original draft, Methodology, Writing – review & editing; **S. Hari Krishna Kumar:** Visualization, Writing – review & editing, Data curation; **Ragothaman Prathiviraj:** Visualization, Writing – review & editing, Data curation; **George Seghal Kiran:** Visualization, Writing – review & editing; **Joseph Selvin:** Supervision, Administration, resource, Writing – review & editing; **Laurent Dufossé:** Supervision, Conceptualization, Writing – original draft, Writing – review & editing.

## Declaration of generative AI and AI-assisted technologies in the writing process

During the preparation of this work, the authors used ChatGPT and Quilbot for paraphrasing and language improvement. Subsequently, they carefully reviewed and edited the material as necessary.

## Data Availability

NCBIStreptomyces bacteria isolated from wetland sediments (Original data).
